# An innovative method for soil vapor extraction to improve extraction and tail gas treatment efficiency

**DOI:** 10.1038/s41598-022-08734-8

**Published:** 2022-04-20

**Authors:** Yang Ding, Yuling Zhang, Zhiqun Deng, Hewei Song, Jili Wang, Haizhao Guo

**Affiliations:** 1 Key Lab of Groundwater Resources and Environment, Ministry of Education, Jilin University, Changchun, 130021 China; 2 College of New Energy and Environment, Changchun, 130021 China; 3 Academic Affairs Office of Jilin University, Changchun, 130021 China

**Keywords:** Environmental chemistry, Environmental impact

## Abstract

This study aims to improve soil vapor extraction (SVE) to address its shortcomings in treating halogenated hydrocarbon-contaminated soil. Indoor simulation experiments based on SVE were conducted to provide technical guidance for the remediation of 1,2-DCA-contaminated soil, with the overall intention of soil repair and ecological restoration. A thermal oxidation SVE (TOSVE) system was designed on the basis of SVE technology for application in the remediation of low-permeability soil contaminated with halogenated hydrocarbons from a chemical plant in Northeast China. Laboratory simulation experiments were conducted based on TOSVE technology to study the removal of target pollutants under different organic contents, moisture and air speeds. For the first time, a new material, scoria, was added to the oxidant at different proportions, and its effect on the exhaust gas treatment efficiency was examined. Thermal extraction improved the extraction efficiency of pollutants from low-permeability soil. Moreover, the adsorption–oxidation effect of 0.1–0.25 mm scoria prepared by 20% Na_2_S_2_O_8_ on 1,2-dichloroethane (1,2-DCA) in tail gas was higher than that of the oxidant without scoria, indicating that scoria is effective in tail gas treatment.

## Introduction

Oil spills are inevitable in the production, refinement, processing, transportation and utilization of oil, as well as in the production processes of related industries. Oil pollutants often enter the soil, causing soil and groundwater pollution^1^. China’s soil has become seriously polluted with petroleum hydrocarbons, and many petroleum hydrocarbon-contaminated sites require urgent remediation. Among the various available physical, chemical and bioremediation technologies, soil vapor extraction (SVE) has attracted considerable attention because of its low cost and good effectiveness^2^. SVE has been shown to be an effective remediation technology to remove volatile organic compounds (VOCs) from unsaturated soils^3^. In the SVE process, fresh air is injected into the contaminated soil through an inlet well, and the negative pressure generated by the vacuum pump and induced draft fan is used to volatilize the oil and subsequently dissolve and adsorb VOCs from the soil. Pollutant gases are pumped to the surface through the pump well for purification before being released into the atmosphere or re-extracted^4^.

SVE has been recognized by the U.S. Environmental Protection Agency (EPA) is one of the most widely used remediation technologies. The success of an SVE project depends on several parameters, including contaminant properties (such as vapor pressure and solubility), soil properties (such as natural porosity and permeability), organic matter and water contents, and operating conditions (such as temperature or airflow rate)^5–10^. High porosity and high permeability are beneficial to the success of SVE, mainly because they have a positive effect on the airflow rate through the soil^2^. Highly soluble pollutants and soils with high water and organic matter contents are unfavorable for SVE, and thus, it is difficult to demonstrate an improvement to the soil pollution situation using SVE in these soils^11,12^. Because soil pollution remediation is a relatively recent process in China, many remediation technologies are still in the laboratory experiment stage, and there has been a lack of systematic research. In particular, the problem of site remediation in low-permeability aerated soils contaminated by volatile halogenated hydrocarbons has not yet been sufficiently explored.

An indoor remediation simulation experiment applying thermal oxidation SVE (TOSVE) technology to remediate contaminated soil in the low-permeability vadose zone was conducted. The main target pollutant for remediation was 1,2-dichloroethane (1,2-DCA), which was detected to be the highest content pollutant in soil samples. The main objective of this study was to design an improved SVE system that can be used in low permeability vadose soils and determine its optimal operating parameters, as well as to determine the optimal tail gas treatment material combination by mixing volcanic ash with different formulations of oxidants. This integrated treatment system can be applied to more complex and difficult soils polluted by organic volatile pollutants, increasing the removal efficiency of pollutants and making the discharge after treatment harmless.

## Materials and methods

### Experimental set up

**Figure 1:**
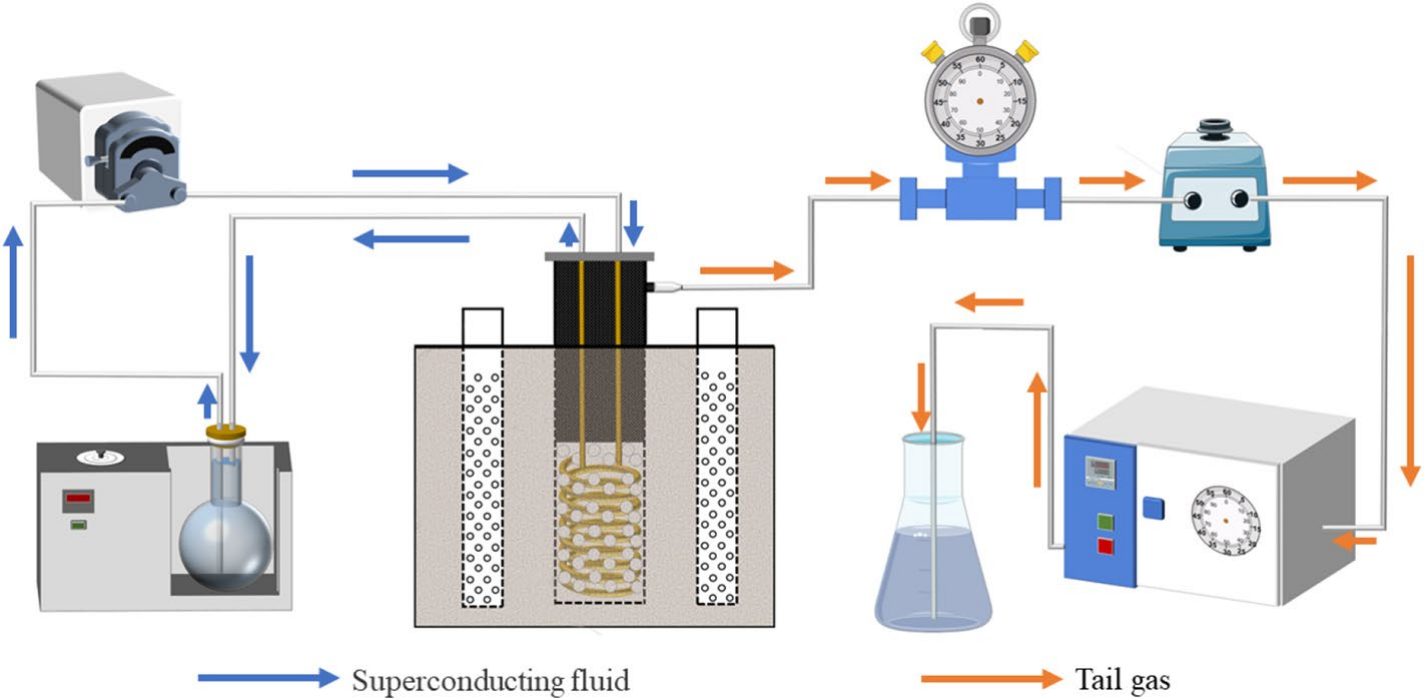
Design of the TOSVE system.

As shown in Fig. 1, contaminated soil samples were placed in a cylindrical plexiglass bucket (height 25 cm, inner diameter 30 cm), and a plexiglass tube (height 20 cm, inner diameter 5 cm) was inserted into the center of the filled soil column as a small simulated extraction well.

Three sampling holes, T1, T2 and T3 (3 cm, 6 cm and 9 cm respectively from the central extraction well), were set on the top cover of the plexiglass bucket for temperature monitoring and soil sample collection, and kept closed during extraction.

### Soil properties

Soil samples were taken from a seriously polluted chemical site in Northeast China, and the sampling depth was concentrated in the silty clay layer of the aerated zone with a burial depth of 2.0–4.0 m. Undisturbed soil samples were taken with a ring knife for the measurement of soil structural properties such as soil bulk density and porosity. The physical properties (bulk density and porosity) and chemical properties (soil pH, organic matter content and mineral composition) of the soil were measured in the laboratory. Soil pH, organic matter content and mineral composition were determined by aluminum box sampling back to the indoor dry ground after self-testing or sent for testing (shown in Table.1). The mineral composition of the soil was analyzed with an X-ray fluorescence spectrometer. When the soil samples were tested, 2 parallel samples were established to ensure the reliability of the experimental results.

**Table 1:** Properties of the soil used in this study.

Unit weight(g/cm^3^)	Moisture content (%)	Porosity (%)	Organic content (%)	pH (%)
1.24	24.27	51.73	8.75	7.42

International standard methodologies were used for the characterization of the prepared and real soils.

**Figure 2:**
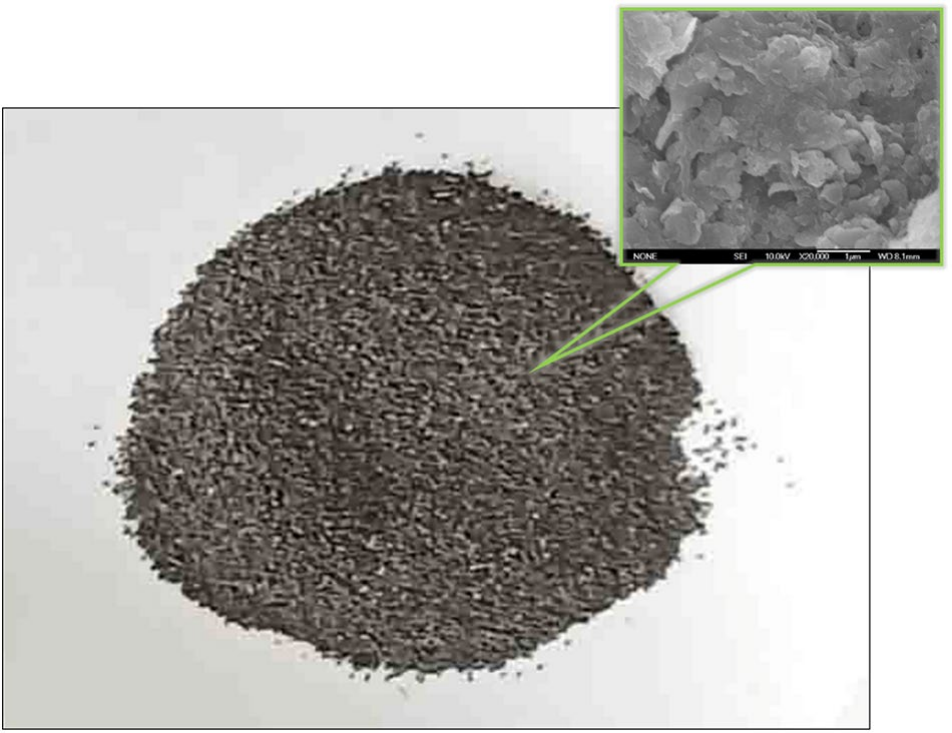
Photograph and SEM image of scoria.

Based on the analysis of the peak spectrum of X-ray diffraction (XRD-6000, Shimadzu, Japan) (Fig. 2), the mineral composition and percentage content of the soil sample were obtained, as shown in Table 2.

**Table 2:** Mineral composition of soil.

Soil	Quartz	Calcite	Dolomite	Feldspar	Clay and amorphous state
Silty clay	23%	3%	2%	8%	63%

The VOC contents in soil samples were determined by gas chromatography-mass spectrometry (Thermo Trace/ISQ) equipped with an automatic scavenging and trapping system (Tekmar Atomx)^13^. Further quantitative analysis of the pollutants in the clay layer of the vadose zone revealed that the main pollutant exceeding the standard was 1,2-DCA, with a detection rate of up to 71%.

Samples with VOC contents in the soil initially determined to be less than 200 μg/kg during the sampling process were placed into 40-mL brown glass headspace bottles with PTFE film screw caps. A magnetic stirring bar was placed in each bottle, sealed and labeled, and weighing records were recorded. During field sampling, the soil samples with a weight of approximately 5 g were placed into vials. The collected samples were put into an airtight bag, sealed and placed upside down in a low-temperature freezer and then sent back to the laboratory for testing as soon as possible (three parallel samples were taken from each sampling point).

### Scoria

The volcanic scoria selected for the experiment is a natural basic volcanic scoria widely found in Northeast China, photographed by PowerShot G7X Mark III (Canon) and JSM-6700F scanning electron microscope (JEOL) (Fig. 2). Natural mineral material scoria is volcanic fine-grained clastics of loose sediments, mainly composed of silicon, iron, manganese, aluminum and other minerals. It is generally dark brown and resistant to acid and alkali weathering, contamination, corrosion and mildew and has pollution-free characteristics. In addition, because of its high porosity, it is lightweight, has a high specific surface area and is often used as an industrial water and drinking water filter material.

A range amount of scoria was added to a glass beaker, ultrapure water was added, and the mixture was stirred with a glass rod. It was then washed several times until the mud composition in the water was washed away, filtered and dried at 105 °C for 2–4 h until completely dry. The dried scoria was sifted to give particle size ranges of 0.1–0.25, 0.25–0.5 and 0.5–1.0 mm.

### Oxidation filler

Scoria (5%, 10% and 20%) were added to the tail gas treatment agent composed of a and B, respectively. After weighing the oxidant dissolved in a small amount of ultrapure water and evenly spraying it on the surface of scoria particles, the mixture was stirred well, air-dried and placed in the interior of the well, and the antioxidant was allowed to effectively adhere to the surface of scoria particles. A certain mass percentage of adsorbed oxide material was thus obtained.

### Experimental design

We conducted normal-temperature SVE experiments under different conditions^14^. Different soil water contents, pumping rates and organic matter contents of the extraction efficiency was tested. On this basis, we then selected the best experimental conditions at different sampling distances and sampling depths.

The conventional SVE experiment was conducted at room temperature (18.0 ± 1.0 °C), the test soil was completely dried and screened silty clay, and the 1,2-DCA concentration was 20 mg/kg. The extraction method was an intermittent extraction method (10 min after each 170 min), and the sample concentration was tested during the intermittent period.

Conventional SVE experiments and TOSVE experiments were conducted to investigate the optimal factor level considering the water content, pumping rate and organic matter content. After the experiment, the influence of different levels of the next factor on the extraction efficiency was investigated on the basis of the current optimal factor level. The horizontal settings of the three factors are shown in Table 3.

**Table 3:** Experimental design of the SVE and TOSVE experiments.

Influence factor	Control method	
Moisture	%	The amount of ultrapure water added
	5	
	10	
	15	
	20	
	25	
Extraction speed	L/min	Adjust the vacuum pump rate
	1	
	2.5	
	5	
Organic content	%	Heat in muffle oven/Add humic acid
	2.26	
	4.45	
	8.32	

After determining the levels of the three influencing factors under the optimal gas phase extraction efficiency at room temperature, the differences in soil extraction efficiency at different locations in the soil were investigated. This involved determining the SVE restoration at different soil sampling depths (5.0 ± 1.0, 12.5 ± 1.0 and 20 ± 1.0 cm) and at different soil sampling distances from the outer wall of the central extraction well (3, 6 and 9 cm).

According to the relevant conclusions obtained from the conventional SVE experiments, the influencing factors of heat conduction enhancement SVE experiments were selected. The increase in moisture content and organic matter removal effect is nonlinear because when the temperature changes, the moisture content of the soil organic matter content may also change, and the increase in the organic matter content of organic matter removal usually has an inhibitory effect. Therefore, the TOSVE experiment focused on the influence on moisture content for the repair, and we did not consider the effect of organic matter content. The concentration of the target pollutants was based on the background value of the contaminated site and the water solubility of pollutants, taken as 20 mg/kg, and the organic matter content was basically the same as that of the contaminated site, which was set at 8.32%.

To study the influence of scoria particle size, oxidant type and ratio on the removal of extracted gas, the prepared scoria adsorption and oxidation material was added to the extraction well, the subsequent experimental device was connected to seal the device, and then the extraction experiment was started.

The TOSVE experimental test considered three different scoria particle size ranges (0.1–0.25, 0.25–0.5 and 0.5–1.0 mm), and the central heating well was set to 80 °C after the start of the experimental device. The concentration of gas extraction was monitored to analyze the impact of different scoria particle sizes on the extraction gas adsorption removal and thus determine the optimal scoria particle size. Next, Na_2_S_2_O_8_ and KMnO_4_ with mass percentages of 5%, 10% and 20% were added to the scoria to act as adsorptive oxidation materials and consider the treatment efficiency based on the tail gas.

## Results

### Factors affecting the efficiency of SVE

**Figure 3:**
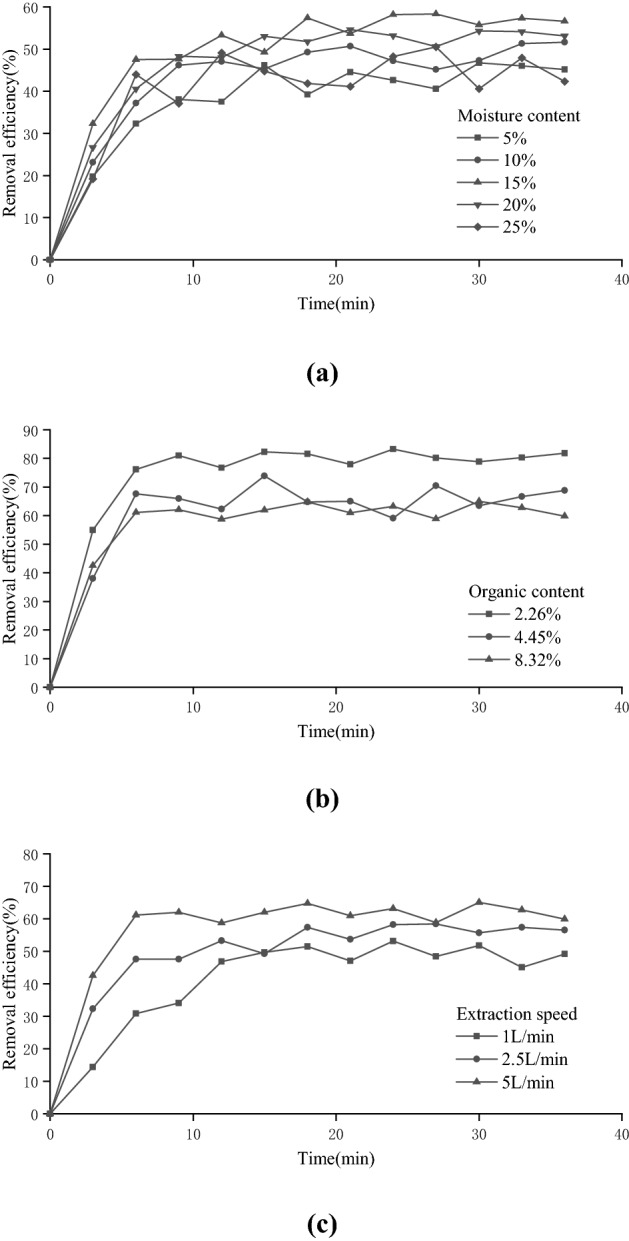
Factors affecting conventional SVE. (**a**) Moisture content, (**b**) Organic matter content, (**c**) Extraction speed.

The removal rates of 1,2-DCA contamination from the soil by SVE under different water content conditions are shown in Fig. 3a. As the extraction time increased, the removal rate of 1,2-DCA under all experimental conditions increased rapidly at first and then gradually stabilized. The time of stable removal rate was 9–15 h, and the overall removal rate was relatively low, roughly in the range of 40%–60%. This was mainly because the permeability of cohesive soil is poor, which is not conducive to the formation of gas channels and cannot effectively promote the migration and transformation of organic matter, making the removal of organic matter difficult. Besides, higher organic matter contents making it harder to remove pollutant. Furthermore, because the organic compounds cannot be completely evenly distributed, the removal rate will naturally fluctuate with time, but the overall trend does not change, similar to the situation at the actual contaminated site. A comparison of the 1,2-DCA removal under different water content experimental conditions shows that removal was best at 15% water content, with a mean value of 56.74% during the stable period (18–36 h) and a peak value of 58.37%.

Some soil pores are closed and thus have no effect on the migration of organic matter, and some are effective pores, which have the possibility of forming airflow channels. These pores are partially occupied by organic matter or water. At low water contents, the adsorption of 1,2-DCA by clay mineral particles is dominant, which is not conducive to its desorption from the particle surface. As the water content increases, the contact opportunity between 1,2-DCA molecules and water molecules increases. Since water molecules are polar molecules, they can more easily attract 1,2-DCA molecules than nonpolar molecules, and they are carried out in the soil layer with airflow. However, when the water content is high, the effective channel of air circulation will be blocked, and the removal rate of 1,2-DCA will also be reduced.

Figure 3b shows that with increasing pumping rate, the duration of the stable period continuously advanced, and the removal rate of 1,2-DCA also continuously improved. The increase in the pumping rate can effectively improve the removal rate of 1,2-DCA, which is roughly between 45 and 65%. The causes of this experimental phenomenon were similar to those of Experiment (1), suggesting a strong relationship between soil permeability and organic matter content. When the pumping rate was 5 L/min, it began to enter a gentle stable period after approximately 6 h. The average removal rate during the stable period from 18 to 36 h was 62.20%, and the peak value was 65.01%. As the pumping speed increased, the rate of 1,2-DCA migration in the soil (diffusion velocity) increased, promoted the first outward migration in a state of freedom and dissolved organic matter, and as the gas molecular diffusion resistance decreased, the mass transfer rate increased. Second, because the balance changes toward a closer association with soil organic matter, the 1,2-DCA removal rate becomes more stable over time. The removal rate of 1,2-DCA was also improved. When the concentration of organic matter was increased in the advance period of extraction, 1,2-DCA could be easily separated from the water phase and organic matter particles under the action of soil negative pressure. As the extraction continued, the concentration of organic matter decreased, the content of organic matter that could be desorbed decreased, and the removal rate slowed down and stabilized.

Figure 3c shows that the time for the three curves to reach the stable period of removal rate was very similar, at approximately 6 h; however, as organic matter content increased, the removal rate of SVE showed a trend of gradual deterioration. Soils with higher organic matter contents tend to contain more adsorption-active functional groups, and when 1,2-DCA enters such soils, it will be adsorbed. The desorption migration of 1,2-DCA becomes difficult under this relatively strong force, thus reducing the removal rate of SVE. A comparison of the three curves reveals that under the low organic matter content condition (2.26%), the removal rate of 1,2-DCA was significantly increased, and the average removal rate reached 80.57% with a peak value of 83.29% during the stable period of 18–36 h. The contact probability of organic matter and 1,2-DCA decreased, and the adsorption strength of organic matter also decreased. Thus, the removal rate of contaminated soil remediation by SVE was improved.

**Figure 4:**
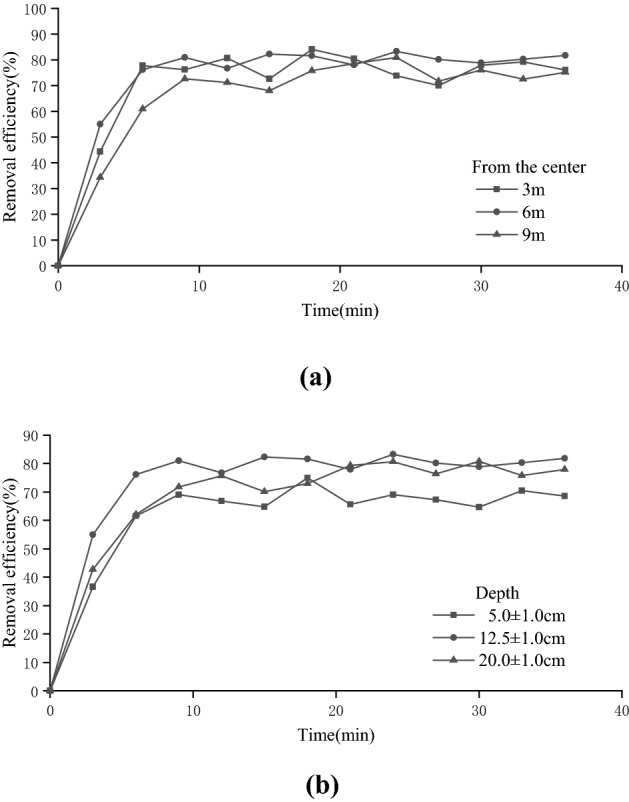
Extraction efficiency of different positions in the experimental device. (**a**) Distance from center of the extraction well, (**b**) Depth.

Figure 4a shows the removal rates of 1,2-DCA from contaminated soil by SVE at different sampling distances under the experimental conditions in which the 1,2-DCA concentration was 20 mg/kg, the water content was 15%, the organic matter content was 2.26%, the pumping rate was 5 L/min and other influencing factors remained unchanged.

**Figure 5:**
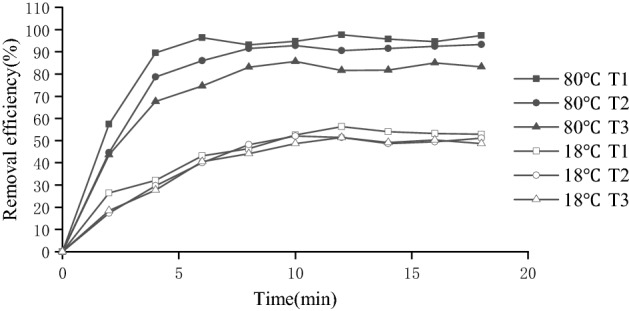
Pollutant extraction efficiency of thermally enhanced SVE.

According to the curve in Fig. 5a, when the sampling depth was 12.5 ± 1.0 cm, the 1,2-DCA removal rate increased rapidly with the extraction time and later stabilized. After reaching the stable period, there was little difference in the removal rate of 1,2-DCA at different sampling distances. The average removal rates were 77.34%, 80.57% and 75.83% at distances of 3, 6 and 9 cm from the outer wall of the extraction well, respectively. The peak value was 84.11% at a sampling distance of 3 cm, and the corresponding residual stock of 1,2-DCA was 3.18 mg/kg. As the extraction time increased, the closer the sample was to the center of the extraction well, the more likely stabilization was to occur more rapidly. At distances of 3 cm and 6 cm, the distance between the two sampling point locations was different, but the repair curve changed, suggesting some influence of the position of the gas injector. At a 6 cm distance from the extraction well wall, the location of the gas injector was closer to the air circulation, and rapid removal of 1,2-DCA was promoted. In addition, the recovery curve at the sampling point 9 cm away from the outer wall of the extraction well took a long time to reach stabilization. The sampling point was far from both the extraction well and the gas injection well and was relatively lagged under the influence of the extraction flow; thus, the time to reach stabilization was prolonged.

Figure 4b shows the removal rates of 1,2-DCA from contaminated soil by SVE at different sampling depths 6 cm away from the central extraction well under the experimental conditions in which the concentration of 1,2-DCA was 20 mg/kg, the water content was 15%, the organic matter content was 2.26%, the pumping rate was 5 L/min and other influencing factors remained unchanged.

It was found that as the extraction time increased, the removal rate of 1,2-DCA increased rapidly at first, tended to stabilize after 9–12 h, and then gradually entered the trailing period. The overall repair efficiency was approximately 65%–83%, and the removal rate did not increase positively with the sampling depth but increased initially and then decreased. The removal rate was optimal when the sampling depth was 12.5 ± 1.0 cm, and the average removal rate was 80.57% during the stable period from 18 to 36 h. The main reason for the overall change of the curve was that at the early stage of the experiment, the concentration of 1,2-DCA was high and could easily be removed by airflow, showing a rapid increase in the removal rate. However, as the concentration of 1,2-DCA decreased, the repair efficiency decreased and tended to stabilize. The curve changed with depth because as the extraction continued, the organic molecules of 1,2-DCA in the soil column tended to converge to the middle extraction well. According to the design of the extraction well and gas injection well in the experimental equipment, the depth of the mesh distribution of the well was 5–20 cm, and the main area of airflow generation was also within this depth range. However, when the sampling distance was located at the middle depth (12.5 ± 1.0 cm), the airflow density was higher than that at the two sides, and thus, the removal efficiency at the middle depth was higher than that at the two sides. In addition, the recovery efficiency of the sampling site located at the highest sampling depth (20.0 ± 1.0 cm) was slightly higher than that of the sampling site located at the shallow sampling depth (5.0 ± 1.0 cm). This was because of the upward migration of 1,2-DCA under its own volatility as the extraction time increased. Second, the adsorbed or dissolved 1,2-DCA also evaporated as water vapor moved upward. As a result, the 1,2-DCA concentration in the upper layer increased. Thus, under the same extraction conditions, the removal rate of the shallow layer was lower than that of the deep layer.

### Factors affecting the efficiency of TOSVE

As shown in Fig. 5, the temperature increases not only shortened the time needed to reach stabilization but also significantly increased the removal rate of 1,2-DCA for sampling points at different distances, and the mean removal rate of the T1 point (8–18 h) increased by 42.92%.

**Figure 6:**
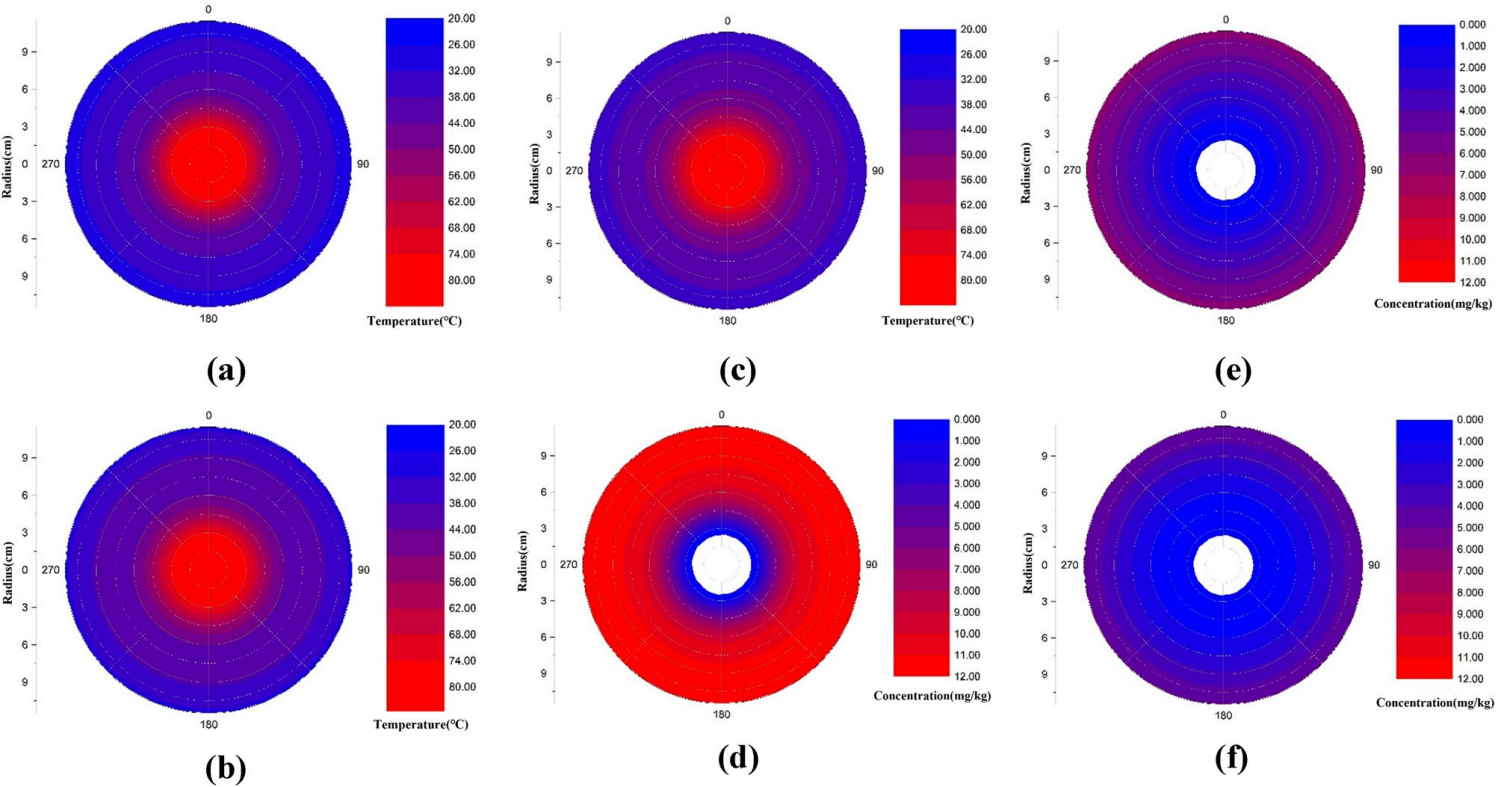
Temperature (**a**–**c**) and concentration (**d**–**f**) simulation.

The temperature field and the concentration field of 1,2-DCA under the optimal experimental conditions were simulated for a circular cross-section with a depth of 12.5 cm under the condition that the central heating well was 80 °C and the moisture content was 15%. The simulation range was a circular area 0–11.5 cm from the center. The circular area of 0–2.5 cm represents the location of the extraction well, and the soil area was between 2.5 and 11.5 cm. The experimental room temperature was approximately 18 °C. Based on the obtained temperature and concentration data, simulations at 2, 4 and 6 h when the temperature and concentration change greatly are shown in Fig. 6.

When the temperature of the central well remained constant at 80 °C, the temperature from the wellbore wall to the surrounding area increased continuously with increasing extraction time from 2 to 6 h (Fig. 6). The closer the central well is, the faster the temperature increase. However, in the marginal area, the temperature of the central well was unchanged, and the temperature remained basically consistent with room temperature.

The concentration field diagrams of 1,2-DCA in Fig. 6d–f show that the 1,2-DCA concentration decreased rapidly over time. When the extraction time reached 2 h, the 1,2-DCA at the edge of the central well wall was largely removed. However, the 1,2-DCA concentration remained high in areas far away from the central well. The effect of the temperature increase on the removal efficiency of 1,2-DCA with a lower boiling point was very clear.

The comparison of the temperature field and concentration field revealed that the temperature increase rate in the experimental soil column was not consistent with the removal rate of 1,2-DCA, and the temperature rise rate was relatively slow. However, with the accumulation of heat, 1,2-DCA with a lower boiling point could be quickly carried away by the airflow, and the temperature rise promoted the desorption and migration of 1,2-DCA. This not only shortened the time required for the removal rate to stabilize but also improved the removal rate of 1,2-DCA.

### The effect of the ratio of scoria and oxidant on tail gas treatment

**Figure 7:**
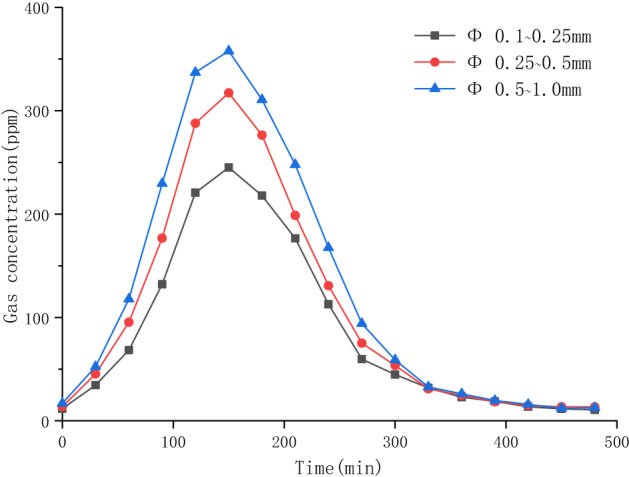
Adsorption effects of different particle sizes of scoria.

Figure 7 shows the effects of variation of the particle size of scoria under the experimental condition of central heating well at 80 °C and no oxidant on pollutant concentrations in the tail gas.

According to the curve in the Fig. 7, as the extraction time increased, the concentration of 1,2-DCA gas increased rapidly at first and then decreased gradually. At a scoria particle size of 0.5–1.0 mm, the peak concentration reached 357.8 ppm, which was still lower than the peak concentration of gas without scoria under the same conditions (466.6 ppm). This indicates that the pore structure of scoria can adsorb 1,2-DCA gas effectively. As the particle size of scoria decreased, the peak value of the curve decreased, and the lowest peak value was 245.1 ppm, indicating that the capacity of 1,2-DCA adsorbed by scoria was continuously increasing. As a highly porous medium, the large specific surface area of scoria can effectively adsorb organic matter. As the scoria particle size decreased, the specific surface area increased continuously, thus increasing contact with gaseous organic matter, increasing the adsorption capacity of scoria and reducing the peak value of the concentration curve.

### Synergistic repair effect of the scoria and oxidant

**Figure 8:**
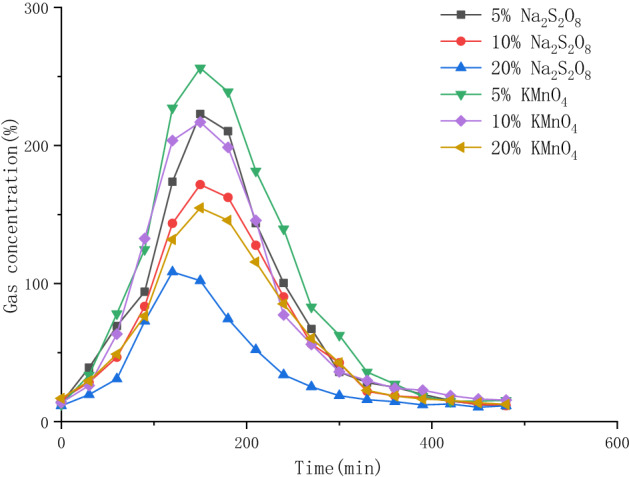
Adsorption effects of different oxidant ratios on the concentrations of 1,2-DCA in TOSVE tail gas.

At 80 °C in the central heating well, the TOSVE experiment was carried out with 0.1–0.25 mm scoria prepared with the two oxidants at three ratios. The experimental results are shown in Fig. 8, and the scattered points were fitted.

Figure 8 shows that all curves initially increased and then decreased, but the amplitude and time of the peak values were different. Under the experimental conditions of 20% Na_2_S_2_O_8_ in scoria, the peak value of extraction gas concentration was 108.4 ppm, and the addition of an oxidant could significantly reduce the concentration of extraction gas. In addition, although the oxidant type varied, the peak concentration clearly decreased with the increase in the mass percentage of oxidants. When the mass of scoria was constant, the increase in oxidants caused more S_2_O_8_^−^ or MnO_4_^−^ to bind to the effective sites on the surface of scoria particles, which can effectively improve the probability of organic matter coming into contact with it. When the gaseous organic matter passes through these sites, an oxidation reaction will occur, thus reducing the concentration in the tail gas.

Moreover, when the mass percentage of the oxidant was constant, the peak concentration of the extraction gas of Na_2_S_2_O_8_ was significantly lower than that of KMnO_4_, and the oxidation of Na_2_S_2_O_8_ was better than that of KMnO_4_ (Fig. 8). This effect is mainly related to the experimental temperature. Increased temperature has been shown to improve the formation rate of the excited state sulfate group. It has a strong reactivity, which significantly promotes the oxidation capacity of Na_2_S_2_O_8_. After coming into contact with 1,2-DCA molecules, the excited sulfate group will react to promote the oxidative degradation of the pollutant. Although the temperature increase can promote the oxidation reaction intensity of KMnO_4_, because 1,2-DCA is an alkane organic matter and does not contain available electron pairs, the ability of KMnO4 to oxidize 1,2-DCA is poor compared with that of Na_2_S_2_O_8_. Therefore, the effect of Na_2_S_2_O_8_ on the extraction of gas by oxidation is better than that of KMnO_4_.

## Discussion

In conventional SVE experiments, the variation of extraction efficiency under different conditions (organic matter content, water content, extraction rate) was discussed, and it was proved that the three factors all have different effects on the extraction efficiency, which is basically consistent with some recent research conclusions^9,11,13^. Labianca et al.^9^ conducted a full scale SVE remediation and proved that soil texture affects air rate and vapor movement through the ground and, subsequently, the total VOC removal. Qin, et al.^11^ proved that soil texture, organic matter content, water content and air flow rate all have different degrees of influence on SVE efficiency, and the influence trend is consistent with this study. Shi et al.^13^ designed a hierarchical extraction device for benzene-contaminated soil remediation to increase the extraction efficiency for more than 90%. This approach does contribute to the desorption of the target pollutant. However, for chlorinated hydrocarbons with higher boiling points, heating extraction can be more effective. It is also easier to operate for full-scale case applications, and costs can be reduced by inserting heating devices instead of layered extraction.

In the TOSVE experiment, we proved that the increase of heating temperature can significantly improve the extraction efficiency, many researchers have proven this finding through experiments^15–17^. Lundin et al. ^18^ found that low heating temperature is not conducive to the removal of pollutants, and the extraction efficiency is significantly improved by increasing the heating temperature, which is consistent with our research conclusions. However, it is worth considering that too high heating temperature will dramatically increase the energy consumption of extraction, which has to be considered in practical engineering. Therefore, we believe that the heating temperature should not exceed 100 ℃, which not only ensures the extraction efficiency but also improves the output/input ratio in practical application.

In the extraction stage, pollutants are volatilized from the soil and transported by carrier gas to the exhaust gas treatment system. The main components of waste gas include carrier gas, a small amount of air, gaseous pollutants, steam, pyrolysis products of soil organic matter and soil particles. If these gaseous pollutants are not effectively removed, secondary pollution will occur. Therefore, the tail gas treatment stage should be another focus of the research. As a mature technology, thermal combustion is easy to operate, occupies little space and has high exhaust gas treatment efficiency^19,20^. However, this method lacks effective safety guarantee and generates new pollutants such as SO2, HCl, NOX. Other researchers have tried to avoid these problems by using Catalytic combustion, but other types of pollutants are inevitably generated^21–23^. Biodegradation is highly praised by many researchers due to its harmlessness and energy saving^24,25^. However, the processing efficiency of this method is low and it has certain requirements for external conditions^20^. Solid adsorption uses porous media to adsorb pollutants by physical or chemical means, and its treatment efficiency depends on the selection of materials and subsequent waste treatment^20,26^. This method is efficient and easy to use, but the price of the adsorbent material affects the feasibility of practical applications^27,28^. In this research, Solid adsorption and liquid oxidation are combined effectively. Firstly, volcanic slag, a common porous material in nature, is selected as the solid adsorption medium, and the oxidant of the target pollutant is added into the volcanic slag. By means of adsorption-chemical reaction, the contact efficiency between oxidizer and harmful substances in tail gas is improved, and the harmful emissions are greatly reduced.

## Conclusion

In this study, the influence of three factors (soil water content, organic matter content and air flow rate) on the extraction efficiency of SVE was revealed, and the self-designed heating device was added to improve the extraction efficiency of soil pollutants. In the aspect of tail gas treatment, the natural material-scoria and oxidizer are innovatively filled into the tail gas treatment device to improve the efficiency of tail gas treatment.

- Under the soil condition of this study, the SVE efficiency reached about 55% when the soil moisture content was 15%.
- The separation efficiency of pollutants was effectively improved by heating device from soil to more than 60–80%.
- Scoria with smaller particle size has more significant adsorption effect on pollutants in tail gas and improved the oxidation efficiency of pollutants after mixing with oxidizer.
